# (Re)Moving the needle: a retrospective, quasi-experimental assessment of the impact of a treatment guideline on oral antibiotic prescribing for orthopedic infections

**DOI:** 10.1017/ash.2025.52

**Published:** 2025-03-21

**Authors:** Russell J. Benefield, Chanah K. Becker, Julie Gray, Heather Cummins, Laura K. Certain

**Affiliations:** 1 Department of Pharmacy, University of Utah Health, Salt Lake City, UT, USA; 2 Department of Pharmacotherapy, University of Utah College of Pharmacy, Salt Lake City, UT, USA; 3 HCA HealthONE Aurora, Aurora, CO, USA; 4 Yale New Haven Hospital, New Haven, CT, USA; 5 University of Utah School of Medicine, Salt Lake City, UT, USA; 6 Division of Infectious Diseases, University of Utah Health, Salt Lake City, UT, USA

## Abstract

**Objective:**

Despite many reports of similar effectiveness between oral and intravenous antibiotics for bone and joint infections, prescribing practice has been slow to change in the United States. We sought to determine if implementing an intravenous-to-oral treatment guideline could increase prescribing of oral antibiotic regimens at our center.

**Design:**

Retrospective, quasi-experimental study.

**Setting:**

Single US academic medical center.

**Patients:**

Patients with bone or joint infections managed by Infectious Disease providers from September 2020 to December 2022.

**Intervention:**

An intravenous-to-oral treatment guideline for patients with bone and joint infections.

**Methods:**

The prescribing rates of fully oral antibiotic regimens before and after implementation of the guideline were compared. Additionally, variables independently associated with oral antibiotic prescribing were identified by logistic regression.

**Results:**

There were 450 patients included: 213 before and 237 after implementation of the guideline. Oral antibiotic prescribing significantly increased following implementation of the treatment guideline to 59% from 33% of patients (difference 25.8%, 95% CI [16.7%, 34.4%]. In multivariable analysis, the post-intervention phase was associated with a significantly greater likelihood of oral antibiotic prescribing (aOR 2.89 [1.90, 4.45]). Other variables independently associated with oral antibiotic prescribing included male sex (aOR 1.88 [1.20, 2.98]), prosthetic joint infection (aOR 0.29 [0.17, 0.47]), and infection with *Enterobacterales* (aOR 2.86 [1.45, 5.92]), methicillin-sensitive *Staphylococcus aureus* [aOR 0.41 [0.26, 0.65]), or coagulase-negative staphylococci (aOR 0.34 [0.18, 0.62]).

**Conclusions:**

Implementation of a treatment guideline resulted in a significant increase in oral antibiotic prescribing. Antimicrobial stewardship programs should implement similar interventions to improve outpatient antibiotic utilization.

## Introduction

Robust data supports the non-inferiority of oral compared to parenteral antimicrobial therapy for bone and joint infections.^1–9^ Despite this, prescribing practice has been slow to change in the United States.^10,11^ Antimicrobial stewardship interventions that increase prescribing of oral regimens for orthopedic infections have the potential to improve patient safety, quality of life, and cost,^4–7,9,10,12–14^ as well as minimize intravenous (IV) fluid consumption during times of shortage, but there are surprisingly few effective interventions described for orthopedic patients.

In response to emerging literature and to increase physician comfort in choosing oral (PO) antibiotics over IV when feasible, our institution created an IV-to-PO treatment guideline for patients with orthopedic infections in November 2021.^2^ We present here the impact of the IV-to-PO treatment guideline on oral antibiotic prescribing practices.

## Methods

### Study design

This was a single-centered, retrospective, quasi-experimental study that compared outcomes before and after implementation of the treatment guideline. The primary outcome was the proportion of patients managed by the Infectious Disease (ID) Division who were discharged on fully oral antibiotic regimens. Secondary outcomes included hospital length of stay and prescribing rate of individual antibiotics. An additional aim was to identify patient characteristics associated with oral antibiotic prescribing.

Institutional approval for this study, including a waiver of informed consent, was granted by the University of Utah Institutional Review Board under IRB Protocol 00111238.

### Setting

Nearly all patients admitted to our hospital with bone and joint infections are seen by one of the inpatient ID consult services, and the ID team recommends a discharge antibiotic plan. The ID consult services generally consist of an attending, fellow, physician assistant, and pharmacist, with some variability between teams. Twenty attending physicians, up to 8 fellows, and 2 physician assistants staff the consult services to varying extents throughout the year. During the time period of this study, there was no formal outpatient parenteral antimicrobial therapy service nor complex outpatient antimicrobial therapy service separate from the ID consult services, and there was no requirement for antimicrobial stewardship approval of discharge antibiotic regimens at our center.

### Guideline implementation

Routine monitoring of patients discharged on antimicrobials by our ID consult services showed that most patients with bone and joint infections were being discharged on IV antimicrobials when many could have been treated with oral regimens. These data were shared with ID providers in-person at a division meeting and feedback was sought on how to increase oral antibiotic prescribing. The primary barrier to oral antibiotic prescribing voiced was limited familiarity with recent literature. A strong consensus emerged for creating a treatment guideline to assist providers in (1) identifying patients who were good candidates for oral regimens and (2) selecting evidence-based regimens that were informed by our local antibiogram.

The guideline was developed by two Ortho-ID specialists in conjunction with antimicrobial stewardship for use by inpatient ID consulting teams. The guideline recommended preferred and alternative oral therapies for the most common pathogens causing orthopedic infections. (Supplementary Appendix) ID providers were oriented to the guideline by group educational sessions accompanied by a division-wide email. Importantly, oral regimens were encouraged but not mandated, and each ID provider was free to deviate from the guideline at their discretion. The guideline was accessible electronically via our health system’s intranet as well as in a division shared folder. The rate of oral antibiotic prescribing was monitored weekly via statistical process control charts and providers were reoriented to the protocol as indicated, either individually or collectively, when oral antibiotic prescribing rates appeared to be decreasing. This data was shared with the division periodically to maintain provider engagement. No additional stewardship interventions were taken to encourage guideline adherence.

### Data collection

Patients with orthopedic infections managed by the ID division between September 14, 2020 and December 31, 2022 were identified by searching the University of Utah Health Enterprise Data Warehouse for patient encounters associated with surgical procedures performed by the Orthopaedic service. Since many bone and joint infections are managed medically, we also searched for patient encounters with the ICD-10 diagnosis codes of M86XXX (osteomyelitis), T845XXX (infection of prosthetic devices), or M462X (vertebral osteomyelitis). Patients were included if they received at least two consecutive calendar days of IV antibiotic therapy while inpatient, were discharged on an IV or oral antibiotic regimen for a planned total treatment duration ≥14 days for an orthopedic infection, were seen by an ID consult service while inpatient, and had a scheduled ID clinic follow-up visit after discharge. Patients were excluded if ineligible for oral antibiotics per the guideline (*Staphylococcus aureus* or *lugdunensis* bacteremia, concern for endovascular infection, no viable oral therapies due to antibiotic resistance and/or patient allergies, oral administration not feasible, atypical organisms such as mold or mycobacteria), were ≤18 years of age, pregnant, discharged to an outside hospital (including long-term acute care or rehabilitation hospitals), prison, or hospice; left against medical advice; expired prior to discharge; required chronic renal replacement therapy; or if their planned antibiotic treatment duration was ≤7 days after discharge. Inclusion was limited to the first eligible encounter for patients with more than one encounter meeting criteria for inclusion. Patients receiving fully oral combination regimens (eg levofloxacin and rifampin) were considered treated with PO, while patients receiving partially oral combination regimens (eg cefazolin and rifampin) were counted as IV.

Data were collected from the University of Utah Health Enterprise Data Warehouse and by chart review by study personnel (RB, JG, HC) using a standard data collection form. Data extracted from the Enterprise Data Warehouse included patient age, primary hospital service, admission and discharge dates, sex, race, ethnicity, discharge disposition, Charlson comorbidity index, payor, primary language, and zip code of primary residence. Data gathered by chart review included clinical and microbiological indication, antibiotics prescribed at hospital discharge, antibiotic allergies, pregnancy status, ID attending physician, ID service (general vs immunocompromised), substance abuse history, housing status, and surgical procedures for drainage or debridement of infection.

### Statistical analysis

Differences in proportions, and median difference in length of stay, between time periods with 95% confidence intervals were determined. Differences were considered statistically significant if the confidence intervals did not contain zero. No formal power calculation was determined *a priori*; rather a convenience sample of all eligible patients was included. Inferential tests of significance were avoided for descriptive data tables.^15^ Standardized differences were reported as an alternative to visualize differences between cohorts.

Variables independently associated with PO-vs-IV prescribing were identified by logistic regression. In brief, variables associated with oral antibiotic prescribing (*P*-value ≤.1) by univariable analysis were added to the initial model. Variables included in the final model were selected by backward and forward selection based on effect likelihood ratios using alpha = 0.05 and guided by Bayesian information criterion to assess model fit.

All data were gathered completely and no additional procedures were necessary to account for missing data. Data were analyzed using JMP Pro version 17.2.0 (SAS Institute Inc., Cary, NC) and R version 4.4.1 (R Core Team 2024).

## Results

Of 999 inpatient encounters screened, 450 met inclusion criteria. Reasons for exclusion included: indication other than treatment of orthopedic infection (197); discharge to outside hospital, prison, hospice, or against medical advice (107); *Staphylococcus aureus* or *lugdunensis* bacteremia (93); repeat encounter during the study period (44); antibiotic duration less than 2 weeks total or 7 days post-discharge (38), no planned ID follow-up (20); chronic renal replacement (19); no viable oral therapies (11); atypical organism (6); concomitant endovascular infection (6); not seen by ID while inpatient (4); expired (3); antibiotics started as outpatient (1).

The pre-intervention group included 213 patients, and the post-intervention group 237. Baseline characteristics were similar between groups although there were fewer patients with prosthetic joint infections and more with diabetic foot osteomyelitis post-intervention (Table 1). Methicillin-sensitive *Staphylococcus aureus* (MSSA) was the most common organism, approximately 20% of patients were treated for culture-negative infections, roughly half of patients received combination regimens, and nearly one-third had a documented antibiotic allergy.

**Table 1.** Baseline characteristics

	Total (n = 450)	Pre-intervention (n = 213)	Post-intervention (n = 237)	Standardized difference
Sex, Male, n (%)	301 (67)	134 (63)	167 (70)	0.16
Age, mean (SD)	55 (15)	55 (16)	56 (15)	0.04
Race, Caucasian, n (%)	379 (84)	185 (87)	194 (82)	0.14
Ethnicity, Hispanic/Latino, n (%)	34 (8)	13 (6)	21 (9)	0.11
Preferred language other than English, n (%)	22 (5)	9 (4)	13 (6)	0.06
Payor, n (%)				
Private or Workers’ Compensation	195 (43)	86 (40)	109 (46)	0.11
Medicare	179 (40)	86 (40)	93 (39)	0.02
Medicaid	62 (14)	33 (15)	29 (12)	0.09
Uninsured	14 (3)	8 (4)	6 (3)	0.07
IV drug use history, n (%)	50 (11)	29 (14)	21 (9)	0.15
Currently experiencing homelessness, n (%)	24 (5)	11 (5)	13 (5)	0.01
Charlson comorbidity index, median (IQR)	2 (0–5)	2 (0–5)	2 (0–4)	0.06^a^
Immunocompromised, n (%)	32 (7)	15 (7)	17 (7)	0.01
Antibiotic allergy, any, n (%)	141 (31)	72 (34)	69 (29)	0.10
Beta-lactam	81 (18)	43 (20)	38 (16)	0.11
Sulfa	48 (11)	26 (12)	22 (9)	0.10
Distance from primary residence to medical center, miles, median (IQR)	28 (9–157)	32 (10–170)	26 (8–134)	0.09^a^
Indication, n (%)				
Osteomyelitis	148 (33)	68 (32)	80 (34)	0.04
Prosthetic joint infection	119 (26)	70 (33)	49 (21)	0.28
Hardware infection	109 (24)	51 (24)	58 (24)	0.01
Diabetic foot osteomyelitis	65 (14)	23 (11)	42 (18)	0.20
Spondylodiscitis	56 (12)	27 (13)	29 (12)	0.01
Septic arthritis	51 (11)	22 (10)	29 (12)	0.06
Bacteremia	22 (5)	13 (6)	9 (4)	0.11
Surgical intervention for source control, n (%)	393 (87)	189 (89)	204 (86)	0.08
Chronic infection, n (%)	62 (14)	34 (16)	28 (12)	0.12
Site of infection, n (%)				
Foot	93 (21)	35 (16)	58 (24)	0.20
Knee	88 (20)	48 (23)	40 (17)	0.14
Spine	57 (13)	27 (13)	30 (13)	0.001
Hip	49 (11)	24 (11)	25 (11)	0.02
Leg	48 (11)	20 (9)	28 (12)	0.08
Ankle	38 (8)	19 (9)	19 (8)	0.03
Shoulder	31 (7)	16 (8)	15 (6)	0.05
Other	64 (14)	28 (13)	36 (15)	0.06
Causative organism, n (%)				
MSSA	143 (32)	70 (33)	73 (31)	0.04
Streptococci	74 (16)	36 (17)	38 (16)	0.02
Coagulase-negative staphylococci	68 (15)	28 (13)	40 (17)	0.11
Enterobacterales	52 (12)	25 (12)	27 (11)	0.01
Gram-positive anaerobes	41 (9)	18 (8)	23 (10)	0.04
MRSA	31 (7)	17 (8)	14 (6)	0.08
Enterococcus spp.	31 (7)	11 (5)	20 (8)	0.13
Pseudomonas spp.	26 (6)	13 (6)	13 (5)	0.03
Candida spp.	22 (5)	14 (7)	8 (3)	0.15
Gram-negative anaerobes	21 (5)	12 (6)	9 (4)	0.09
Cutibacterium spp.	21 (5)	11 (5)	10 (4)	0.05
Polymicrobial	119 (26)	56 (26)	63 (27)	0.01
Culture negative	89 (20)	36 (17)	53 (22)	0.14
Multi-drug regimen, n (%)	206 (46)	97 (46)	109 (46)	0.01
Discharge Disposition, home, n (%)	333 (74)	149 (70)	184 (78)	0.18
Hospital service				0.16
Orthopedics	192 (43)	98 (46)	94 (40)	0.13
Internal medicine	188 (42)	82 (39)	106 (45)	0.13
Plastic surgery	24 (5)	13 (6)	11 (5)	0.07
Other	46 (10)	20 (9)	26 (11)	0.05

Abbreviations: SD, standard deviation; IQR, interquartile range; MRSA, methicillin-resistant *Staphylococcus aureus*; MSSA, methicillin-susceptible *Staphylococcus aureus.*

a Estimated by Cliff’s delta due to non-parametric distribution.

**Figure 1.**
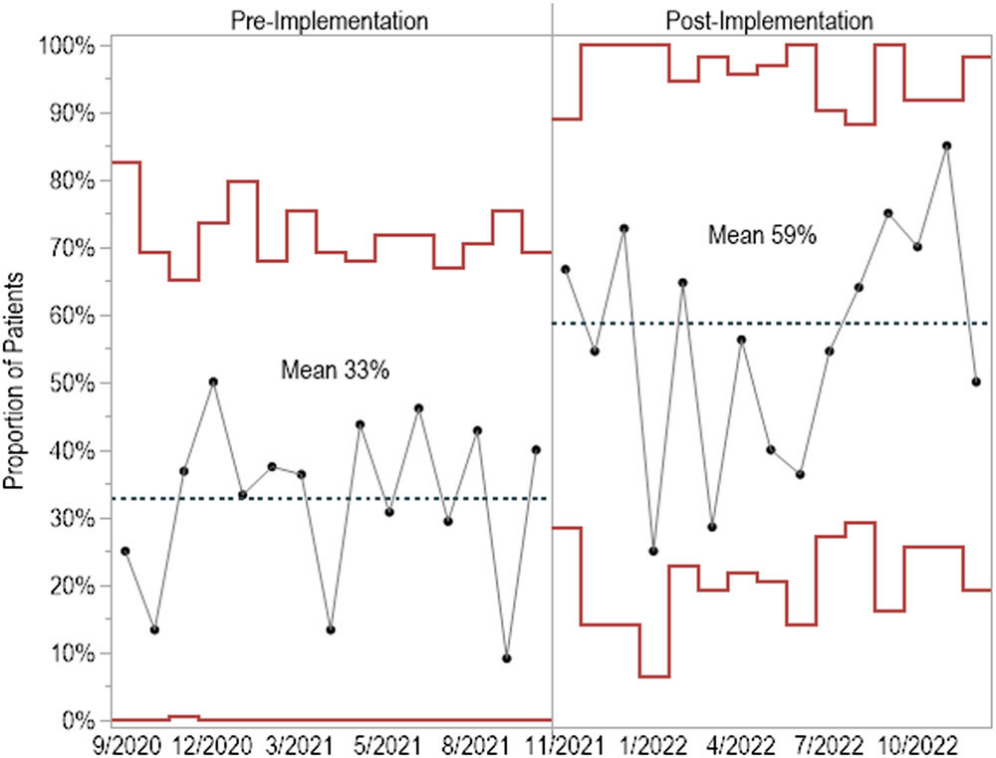
P-chart of the proportion of patients prescribed fully oral antibiotic regimens at discharge before and after implementation of the treatment guideline. Each circle represents the observed proportion of patients in each 4-week interval prescribed oral regimens. The center line (mean, dotted) and surrounding upper and lower control limits (solid red) are plotted for each time period. The number of patients included in each time interval varied from 8 to 25 and averaged 15.

**Figure 2.**
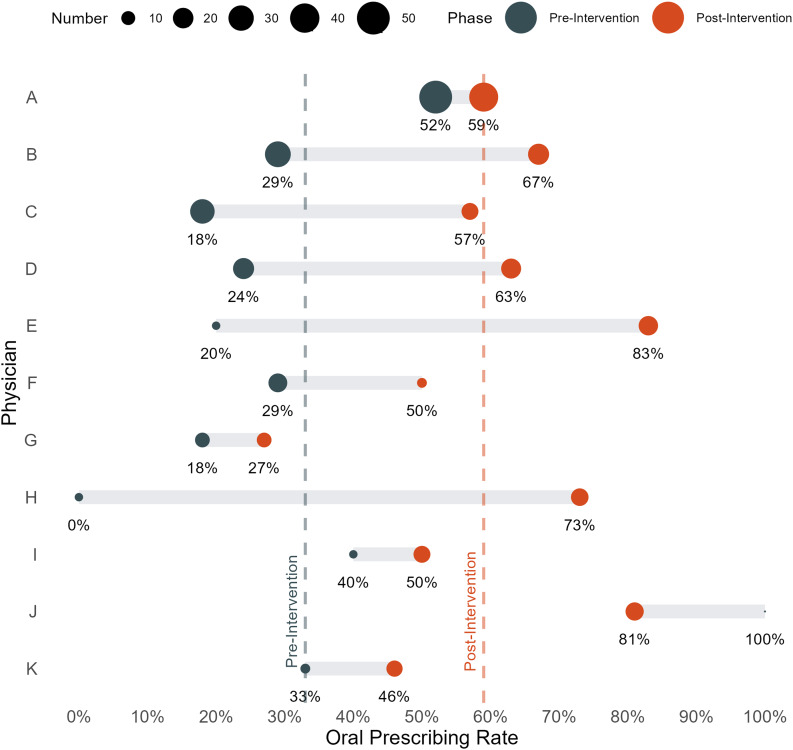
Dumbbell plot of oral antibiotic prescribing rate during the pre-intervention (blue) and post-intervention (orange) time periods by attending ID physician. The size of each dot is proportional to the number of patients treated during each intervention phase. The vertical dashed lines show the overall prescribing rates for each time period. The plot is limited to the attending physicians who managed the greatest number of patients.

Prescribing of oral antibiotics significantly increased following implementation of the treatment guideline to 59% from 33% of patients (difference 25.8%, 95% CI [16.7%, 34.4%] (Figure 1). The most common oral antibiotics included fluoroquinolones (38%), penicillins (36%), trimethoprim-sulfamethoxazole (23%), and tetracyclines (23%), while the most common IV antibiotics included cephalosporins (58%) and vancomycin (36%). Independent of route, when compared to the pre-intervention phase there was more frequent prescribing of penicillins (28% vs 16%, difference 12.3%, [4.6%, 19.7%]), trimethoprim-sulfamethoxazole (16% vs 5%, difference 10.4%, [4.8%, 15.9%]), and tetracyclines (16% vs 7%, difference 9.0%, [3.0%, 14.7%]) and less frequent prescribing of cephalosporins (26% vs 42%, difference –16.5%, [–7.8%, –25.0%]) and vancomycin (16% vs 23%, difference –7.4%, [–0.04%, –14.8%]) post-intervention. Fluoroquinolone prescribing did not significantly change following guideline implementation (21% vs 22%, difference 1.0%, [–8.6%, 6.6%]). The P-chart demonstrated considerable process variability in both intervention phases, which correlated with providers that were in service at the time. While there was variability in the extent to which each attending physician shifted their practice, nearly all physicians prescribed oral regimens with greater frequency after implementation of the guideline (Figure 2).

When accounting for other variables significantly associated with PO-vs-IV prescribing, the post-intervention phase was associated with a significantly greater likelihood of oral antibiotic use. (Table 2) Other variables independently associated with PO prescribing included sex and infection with *Enterobacterales*. Oral prescribing was less likely for patients with prosthetic joint infections, or infections with MSSA or coagulase-negative staphylococci.

**Table 2.** Univariate and multivariate associations with oral antibiotic prescribing

	Total (n = 450)	IV (n = 242)	Oral (n = 208)	Crude		Adjusted
				OR (95% CI)	*P*-value	aOR (95% CI)
Sex, Male, n (%)^a^	301 (67)	143 (59)	158 (76)	2.19 (1.45–3.29)	.0001	1.88 (1.20–2.98)
Age, mean (SD)^a^	55 (15)	58 (15)	52 (15)	0.98 (0.96–0.99)	.0002	
Race, Caucasian, n (%)	379 (84)	210 (87)	169 (81)	0.66 (0.40–1.10)	.11	
Ethnicity, Hispanic/Latino, n (%)^a^	34 (8)	13 (5)	21 (10)	1.98 (0.96–4.06)	.06	
Preferred language other than English, n (%)	22 (5)	9 (4)	13 (6)	1.73 (0.72–4.12)	.21	
Payor, n (%)^a^						
Private or Workers’ Compensation	195 (43)	94 (39)	101 (49)	Ref		
Medicare	179 (40)	117 (48)	62 (30)	0.49 (0.32–0.75)	.0008	
Medicaid	62 (14)	28 (12)	34 (16)	1.13 (0.64–2.02)	.68	
Uninsured	14 (3)	3 (1)	11 (5)	3.41 (1.03–15.43)	.05	
Immunocompromised ID service	32 (7)	21 (9)	11 (5)	0.59 (0.28–1.25)	.16	
IV drug use history, n (%)^a^	50 (11)	20 (8)	30 (14)	1.87 (1.03–3.41)	.04	
Currently experiencing homelessness, n (%)^a^	24 (5)	9 (4)	15 (7)	2.01 (0.88–4.88)	.10	
Charlson comorbidity index, median (IQR)	2 (0–5)	2 (0–5)	2 (0–4)	0.97 (0.91–1.04)	.52	
Antibiotic allergy, any, n (%)	141 (31)	82 (34)	59 (28)	0.77 (0.52–1.16)	.21	
Beta-lactam	81 (18)	50 (21)	31 (15)	0.67 (0.41–1.10)	.11	
Sulfa	48 (11)	30 (12)	18 (9)	0.67 (0.36–1.24)	.20	
Distance from primary residence to medical center, miles, median (IQR)	28 (9–157)	32 (10–158)	25 (8–144)	1.00 (0.99–1.00)	.20	
Indication, n (%)						
Osteomyelitis^a^	148 (33)	65 (27)	83 (40)	1.81 (1.22–2.69)	.003	
Prosthetic joint infection^a^	119 (26)	91 (38)	28 (13)	0.26 (0.16–0.42)	<.0001	0.29 (0.17–0.47)
Hardware infection	109 (24)	58 (24)	51 (24)	1.03 (0.67–1.59)	.89	
Diabetic foot osteomyelitis^a^	65 (14)	22 (9)	43 (21)	2.61 (1.50–4.53)	.0005	
Spondylodiscitis	56 (12)	34 (14)	22 (11)	0.72 (0.40–1.27)	.26	
Septic arthritis	51 (11)	24 (10)	27 (13)	1.35 (0.76–2.43)	.31	
Bacteremia	22 (5)	9 (4)	13 (6)	1.73 (0.73–4.26)	.21	
Surgical intervention for source control, n (%)^a^	393 (87)	218 (90)	175 (84)	0.58 (0.33–1.02)	.06	
Chronic infection, n (%)^a^	62 (14)	41 (17)	21 (10)	0.55 (0.31–0.97)	.03	
Causative organism, n (%)						
MSSA^a^	143 (32)	94 (39)	49 (23)	0.49 (0.32–0.73)	.0005	0.41 (0.26–0.65)
Streptococci^a^	74 (16)	29 (12)	45 (22)	2.03 (1.22–3.37)	.006	
Coagulase-negative staphylococci^a^	68 (15)	47 (19)	21 (10)	0.47 (0.26–0.80)	.005	0.34 (0.18–0.62)
Enterobacterales^a^	52 (12)	14 (6)	38 (18)	3.64 (1.91–6.93)	<.0001	2.86 (1.45–5.92)
Gram-positive anaerobes^a^	41 (9)	15 (6)	26 (13)	2.16 (1.11–4.20)	.02	
MRSA	31 (7)	15 (6)	16 (8)	1.26 (0.61–2.62)	.53	
Enterococcus spp.	31 (7)	16 (7)	15 (7)	1.10 (0.53–2.28)	.80	
Pseudomonas spp.^a^	26 (6)	9 (4)	17 (8)	2.30 (1.00–5.28)	.04	
Candida spp.	22 (5)	12 (5)	10 (5)	0.97 (0.41–2.29)	.94	
Gram-negative anaerobes^a^	21 (5)	6 (2)	15 (7)	3.06 (1.22–8.71)	.02	
Cutibacterium spp.	21 (5)	10 (4)	11 (5)	1.30 (0.54–3.11)	.56	
Polymicrobial^a^	119 (26)	52 (21)	67 (32)	1.74 (1.14–2.65)	.01	
Culture negative	89 (20)	44 (18)	45 (22)	1.24 (0.78–1.98)	.36	
Multi-drug regimen, n (%)	206 (46)	114 (47)	92 (44)	0.89 (0.61–1.29)	.54	
Hospital service^a^						
Orthopedics	192 (43)	114 (47)	78 (38)	Ref		
Internal medicine	188 (42)	88 (36)	100 (48)	1.66 (1.11–2.049)	.01	
Plastic surgery	24 (5)	15 (6)	9 (4)	0.88 (0.37–2.10)	.77	
Other	46 (10)	25 (10)	21 (10)	1.23 (0.64–2.35)	.53	
Post-intervention phase^a^	237 (53)	99 (41)	138 (66)	2.85 (1.94–4.19)	<.0001	2.89 (1.90–4.45)

Abbreviations: aOR, adjusted odds ratio; CI, confidence interval; IQR, interquartile range; IV, intravenous; MRSA, methicillin-resistant *Staphylococcus aureus*; MSSA, methicillin-susceptible *Staphylococcus aureus*; OR, crude odds ratio; SD, standard deviation.

a Variable added to initial multivariable model.

A greater proportion of patients prescribed oral antibiotic regimens were discharged home than to a facility (85% vs 65%, difference 19.7%, 95% CI [11.8%, 27.3%]. Hospital length of stay was shorter in patients discharged on PO compared to IV (4.88 vs 5.68 days, median difference 0.77 days, [–0.04, 1.38]), although this was not statistically significant and based on chart review was most likely not related to antibiotic choice.

## Discussion

In this study of antibiotic prescribing for bone and joint infections at a US medical center, implementation of a treatment guideline that outlined when and how to use PO antibiotics was associated with a significant shift from IV to PO prescribing. This effect was immediate and sustained over the observation period, indicating a relatively simple intervention that required limited effort to maintain. This observation is consistent with the well-known benefits of IV-to-PO treatment guidelines as antimicrobial stewardship tools.^16^ Importantly, the practice change appeared to be widely adopted and not driven by a handful of providers. While we did not assess safety outcomes in this work, nearly all patients discharged on oral regimens were able to avoid central lines and the inherent risk of vascular access complications.^5,12,14^ Greater use of oral antibiotics likely also resulted in greater patient satisfaction and reduced health care costs based on what others have reported.^5–7,9,10,13^

Our findings have the inherent limitations of a retrospective observational design: potential for confounding and inability to assess causality. While we attempted to control for confounding through logistic regression there may be unexplained confounders that contributed to our observations. Additionally, our single-center experience may not apply to other centers with different patient populations or practice models. Relatively few patients were included with methicillin-resistant *Staphylococcus aureus* infections, and this remains a significant gap in the existing literature.

While in aggregate the guideline was associated with increased prescribing of oral regimens, the considerable variability observed in prescribing warrants further investigation. For example, identifying why patients with prosthetic joint or staphylococcal infections were less likely to receive oral antibiotics may lead to further opportunities for improvement.

In conclusion, implementation of an IV-to-PO guideline resulted in a significant increase in PO antibiotic prescribing. Antimicrobial stewardship programs should implement similar interventions to improve outpatient antibiotic utilization.

## Supporting information

Benefield et al. supplementary material Benefield et al. supplementary material

## Data Availability

Deidentified data is available from the authors upon request.

## Supplementary material

For supplementary material accompanying this paper visit http://doi.org/10.1017/ash.2025.52. click here to view supplementary material
