# Supplementary material for: (Re)Moving the needle: a retrospective, quasi-experimental assessment of the impact of a treatment guideline on oral antibiotic prescribing for orthopedic infections
Source: Antimicrob Steward Healthc Epidemiol. 2025 Mar 21;5(1):e84. doi: 10.1017/ash.2025.52 (PMC11951238; doi:10.1017/ash.2025.52)
Supplement: Benefield et al. supplementary material [file S2732494X2500052Xsup001.docx]

**Recommended Oral Therapy Regimens**

| Organism | Trimethoprim-Sulfamethoxazole DS (160/800 mg)  2 tabs BID[a][b] | Doxycycline  100 mg BID | Linezolid  600 mg BID[a] | Clindamycin  600 mg TID | Levofloxacin  750 mg daily[a][c]  OR  Ciprofloxacin 750 mg BID[a] | Levofloxacin  750 mg daily[a][c]  OR  Ciprofloxacin  750 mg BID[a]  +  Rifampin  300-450 mg BID | Dicloxacillin 500 mg TID or QID | Amoxicillin/  Clavulanate  875 mg/ 125 mg BID-TID[a] | Amoxicillin 1000 mg TID[a] |
| --- | --- | --- | --- | --- | --- | --- | --- | --- | --- |
| **Gram-positive organisms[e]** | | | | | | | | | |
| MRSA | Preferred | Alternative | Preferred | Alternative | -- | Preferred | -- | -- | -- |
| MSSA | Preferred | Alternative | Alternative | Alternative | -- | Preferred | Alternative | Alternative | -- |
| Coagulase Negative *Staphylococcus* | Alternative[d] | Preferred | Preferred | Alternative | -- | Preferred | Alternative[d] | Alternative[d] | -- |
| *Enterococcus spp* (ampicillin-resistant) | -- | -- | Preferred | -- | -- | -- | -- | -- | -- |
| *Enterococcus spp* (ampicillin-susceptible) | -- | -- | Alternative | -- | -- | -- | -- | Alternative | Preferred |
| *Group A streptococcus* | -- | -- | -- | Alternative | Alternative | **--** | **--** | Alternative | Preferred |
| *Viridans Streptococcus spp* | -- | -- | -- | Alternative | Alternative | **--** | **--** | Alternative | Preferred |
| *C acnes* | -- | Alternative | -- | Alternative | -- | **--** | **--** | Alternative | Preferred |
| **Gram-negative organisms[e]** | | | | | | | | | |
| *E coli* | Preferred | -- | -- | -- | Preferred | -- | -- | Alternative | -- |
| *K pneumoniae* | Preferred | -- | -- | -- | Preferred | -- | -- | Alternative | -- |
| *E cloacae* | Preferred | -- | -- | -- | Preferred | -- | -- | -- | -- |
| *P aeruginosa* | -- | -- | -- | -- | Preferred | -- | -- | -- | -- |
| *Proteus mirabilis* | Alternative[d] | -- | -- | -- | Preferred | -- | -- | Alternative | -- |
| *Serratia marcescens* | Alternative[d] | -- | -- | -- | Preferred | -- | -- | -- | -- |

[a] Renal dose adjustment required

[b] Use with extreme caution in patients on ACE/ARB therapy, especially if diabetic

[c] Levofloxacin is the preferred fluoroquinolone when treating gram-positive organisms due to a higher barrier to resistance and broader gram-positive spectrum

[d] Considered an alternative only when susceptibilities are available

[e] If culture negative or patient has no microbiological data and strong suspicion of infection remains, then empirically select combination regimen that covers both gram-positive and gram-negative organisms, and consider patient history and exposures to determine the need for MRSA/MRSE, *P aeruginosa, and/or atypical* coverage.
